# A novel diagnostic sign of hip fracture mechanism in ground level falls: two case reports and review of the literature

**DOI:** 10.1186/1752-1947-6-136

**Published:** 2012-05-29

**Authors:** Douglas W Kelly, Brian D Kelly

**Affiliations:** 1 5501 North 19th Avenue, Suite #432, Phoenix, AZ, 85015, USA; 2 1919 East Thomas Road, Phoenix, AZ, 85016-7710, USA

## Abstract

**Introduction:**

Most elderly hip fractures are the result of a ground level fall. Defining high risk falls and fracture mechanisms are important to develop successful hip fracture prevention programs. This case series presents a previously unreported diagnostic sign and for the first time documents a hip fracture mechanism for a knee impact injury from a ground level fall in two elderly patients.

**Case presentation:**

Case 1 was a 65-year-old Caucasian woman who fell forward with initial contact to her left knee, sustaining an impacted femoral neck fracture of her ipsilateral left hip. Case 2 was a 92-year-old Caucasian woman who fell bending forward, impacting her left knee and sustaining a comminuted intertrochanteric fracture of her ipsilateral left hip. The fractures occurred as a result of unprotected ground level falls in a forward direction with initial impact to the knee. The knee contusions were located near Gerdy’s tubercle and appear characteristic of a direct impact injury.

**Conclusion:**

The physical finding of a small localized site of impact and/or contusion in the anterior aspect of the knee in both of these patients with radiographic evidence of an ipsilateral hip fracture would strongly suggest that a knee impact injury can transmit enough energy to the proximal femur by axial loading to result in the hip fracture. The physical finding described is a reliable indicator of this hip fracture mechanism.

## Introduction

A simple ground level fall can have serious consequences. More than 90% of hip fractures in the elderly population are the result of a ground level fall [1-4]. Current hip fracture prevention strategies in this age group are based mostly on the prevention of osteoporosis, decreasing the number and severity of falls, and protection of the proximal femur when a fall occurs [5]. Understanding the relationships between fall characteristics and hip fracture mechanisms is critical to developing successful prevention programs.

Investigators studying hip fracture mechanisms have concluded that the most common mechanism of hip fracture in the elderly is a sideways fall with lateral hip impact [6-11]. To date there has been no reliable physical finding or radiographic feature on which to base these conclusions or to define fracture mechanisms objectively.

The cases of two patients presenting with a distinct and previously unreported physical finding indicating a hip fracture mechanism are described. An in-depth review of the literature would suggest that this is not only a characteristic finding, but one of clinical importance.

## Case presentations

### Case 1

**Figure 1:**
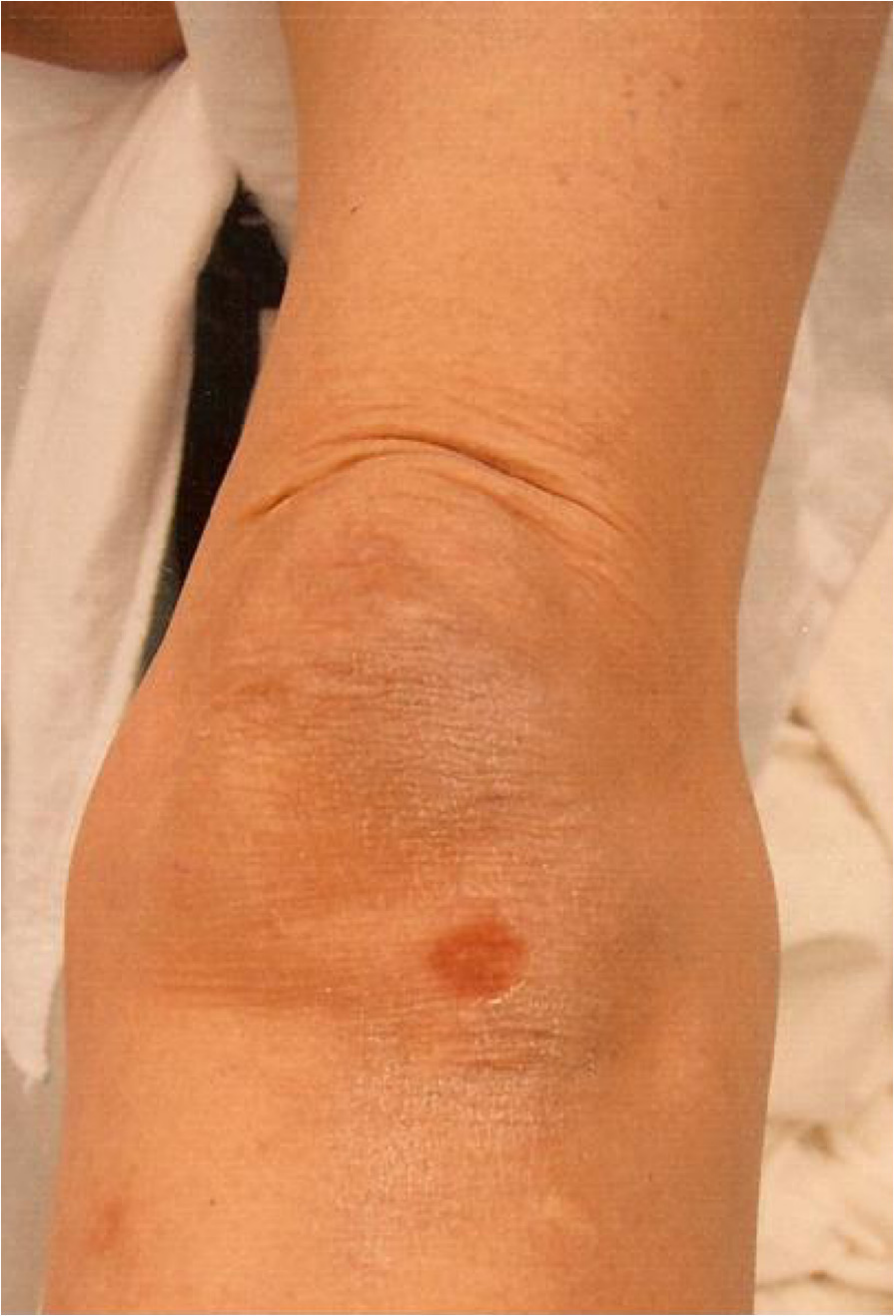
Case 1: photograph of the left knee at the time of initial evaluation showing the area of circular, localized contusion near Gerdy’s tubercle.

**Figure 2:**
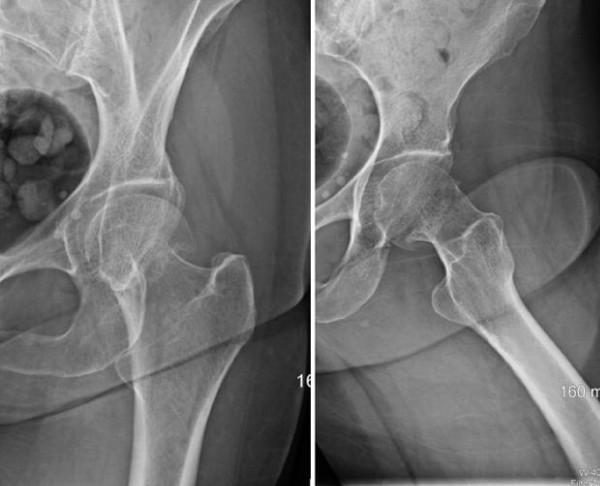
Case 1: preoperative radiographs of the left hip (anteroposterior and lateral views) demonstrating the impacted femoral neck fracture.

A healthy, 65-year-old Caucasian woman fell forward when she tripped over a small stepping stone, landing directly on her flexed left knee. She landed outdoors on a hard stone surface with initial isolated impact on the anterior aspect of her left knee. There was no other site of impact. An eyewitness confirmed this mechanism. She complained of left hip discomfort only. A physical examination showed relevant findings only in her left lower extremity. Her left leg was held in slight increased external rotation with some discomfort on attempted hip range of motion. Her hip showed direct trochanteric tenderness, but no ecchymosis. A distinct area of circular, localized fresh skin contusion was found on physical examination of her ipsilateral left knee, measuring 1 cm to 1.5 cm in diameter at a location proximal and lateral to the tibial tubercle, near Gerdy’s tubercle (Figure 1). The contusion had minimal tenderness. Her knee was without effusion, was stable to ligament examination and had no additional tenderness. The extensor mechanism was intact. Radiographs of her left hip showed a slightly impacted femoral neck fracture (Figure 2). She was treated operatively with an *in situ* cannulated hip screw fixation and healed completely within eight weeks. She returned to full activities without complaints.

### Case 2

**Figure 3:**
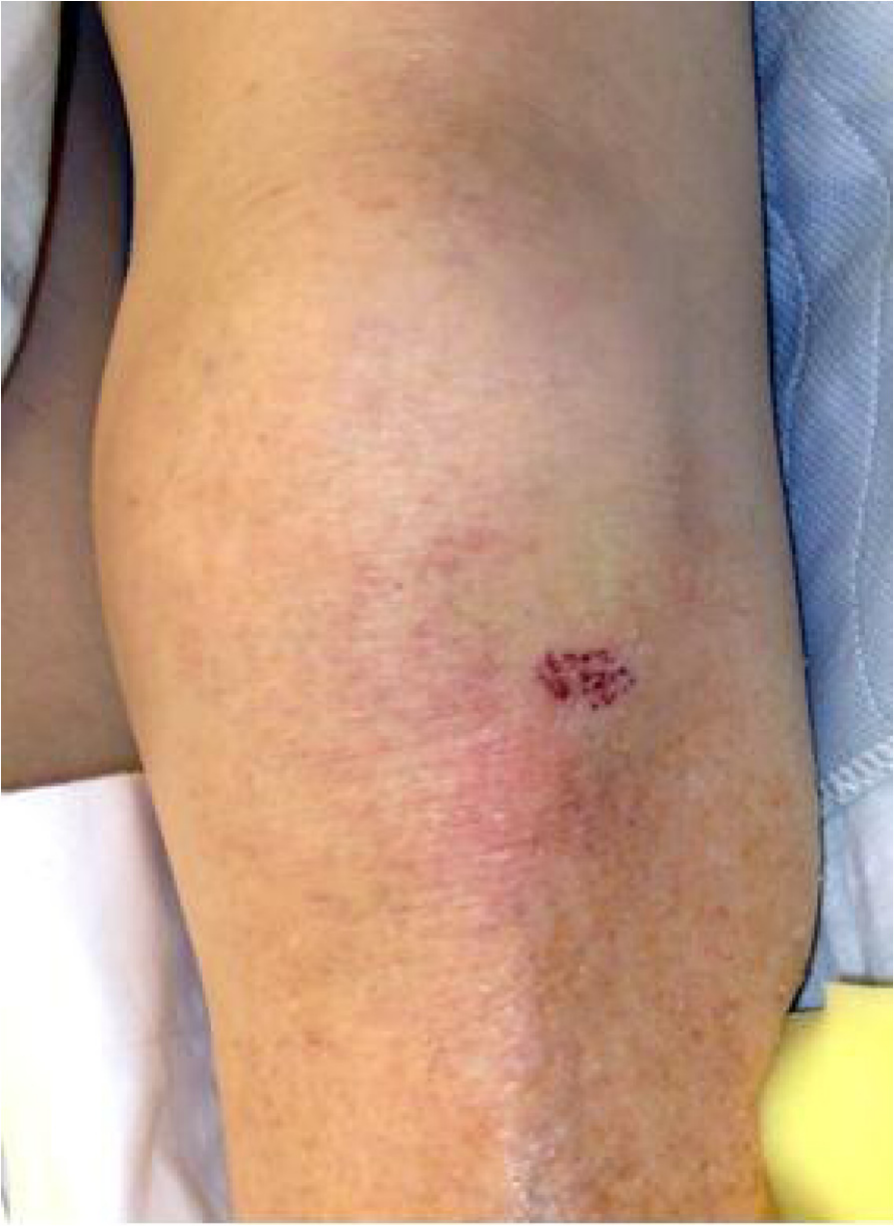
Case 2: photograph of the left knee at the time of initial evaluation showing nearly identical contusion as seen in Case 1.

**Figure 4:**
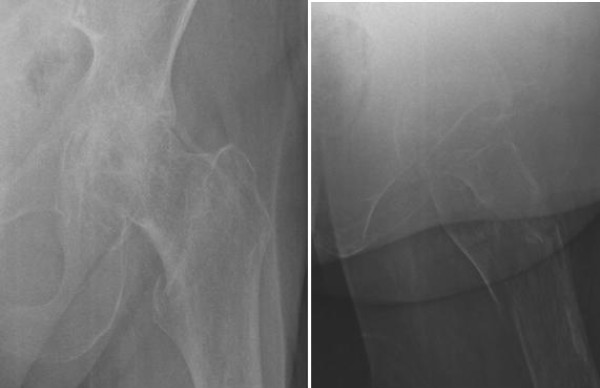
Case 2: preoperative radiographs of the left hip (anteroposterior and lateral views) demonstrating a comminuted intertrochanteric fracture, degenerative arthritis and osteopenia.

A 92-year-old Caucasian woman with chronic atrial fibrillation became lightheaded bending forward and fell indoors on a hard tile surface. She distinctly recalled landing directly on her flexed left knee. She had no other site of impact*.* She reported an initial isolated left knee impact and no other injury. She was unable to ambulate following the fall. She had only left knee complaints when first evaluated in the emergency room and was triaged as a knee injury. Her medical history revealed chronic atrial fibrillation and past hypotensive episodes but without falls. A physical examination showed only left lower extremity findings. Her left lower extremity was held in a position of external rotation and with some shortening. Her left hip showed restriction of motion and discomfort on attempted motion. It was tender over the greater trochanter and showed no ecchymosis. Examination of her ipsilateral left knee revealed a distinct area of circular, localized fresh skin contusion which measured 1 cm to 1.5 cm in diameter at a location proximal and lateral to the tibial tubercle, near Gerdy’s tubercle (Figure 3). The contusion had mild tenderness. A knee examination revealed a 1+ effusion, but no ligament instability, and an intact extensor mechanism. Radiographs (three views of her left knee) showed slight osteopenia and evidence of an effusion, but no other acute findings. Radiographs of her left hip revealed a comminuted intertrochanteric type hip fracture (Figure 4). Operative treatment with a sliding compression hip screw resulted in satisfactory healing and our patient was able to return to ambulation.

The knee contusions on both patients healed without any further treatment and neither had any knee symptoms at follow-up three months later.

## Discussion

Despite frequent falls in the elderly, only about 1% result in hip fracture [3,4]. A hip fracture occurs when the energy absorbed around the proximal femur exceeds a critical threshold [6]. Certain fall characteristics are associated with increased risk of hip fracture. Combinations of biomechanical and clinical fracture studies, fall simulation and clinical fall studies provide important information about these high risk falls and the mechanisms of how fractures occur.

There is general agreement that most elderly hip fractures are the result of a sideways fall and impact occurring near or around the hip [6-10]. No investigator in these studies reported any objective finding to conclude a hip fracture mechanism. They determined the hip area to be the site of impact from the patients’ reported histories, and/or the reports of independent witnesses to the fall. The weaknesses of these studies include the potential for preconceived bias of fall direction and impact location coming from patients dealing with a painful hip condition and the inability of some elderly patients to describe the circumstances of the fall when dealing with a life-threatening, traumatic event [9].

One study did perform physical examinations that included the recording of bruises and hematomas [11]. The association of physical findings with fall mechanics was carefully studied. This study documented the finding of a fresh subcutaneous hematoma in the trochanteric region of the hip in some patients and analyzed lucid patient and independent eyewitness responses. They were able to conclude that the typical hip fracture is the result of a fall and a subsequent impact on the greater trochanter of the proximal femur. The majority (76%) of their patients with hip fractures reported that they had fallen directly to the side. In 56% of these patients, a fresh subcutaneous hematoma was present. Such a hematoma was present in 6% of controls (patients who fell without sustaining a hip fracture).

The physical finding of a subcutaneous hip hematoma is an important objective finding to help document the direction of the fall and the impact area. However, the clinical significance of this physical finding can vary greatly and its documentation has occurred only in this one study [11]. Hematomas in the area of the hip can occur without direct impact in association with extracapsular hip fractures resulting from rotational or indirect trauma. Some patients with a hip fracture and direct impact will show no signs of hematoma; while others will present with a hip hematoma, show no evidence of a hip fracture and have no history of impact [11].

The present series documents two cases of ground level falls in which both patients describe falling directly forward in an unprotected manner, with initial and isolated direct anterior knee impact. Both patients presented with nearly identical, circular and localized anterior knee contusions near Gerdy’s tubercle. Neither patient had any physical finding indicating other impact sites. This is the first description in the literature of a physical finding of this nature and location found in association with an ipsilateral hip fracture in the elderly.

Investigators of ground level falls and hip fracture mechanisms have concluded that direction of fall is a key determinant of a safe landing. Fall direction influences impact sites as well as a series of responses. A sideways fall is associated with a higher risk of hip fracture than a fall in any other direction. It exposes the hip to unusual direct impact landing forces and decreases the chances of knee and hand protective responses to absorb energy. Conversely, falling forward, hitting the hand(s) and/or the knee(s), seems to ensure a safer landing [6-10]. Despite this evidence, a ground level fall in a forward direction accounts for nearly 8% to 10% of all hip fractures [11]. The mechanism of hip fracture in these cases has never been documented.

The only mechanism of injury to reasonably account for the hip fractures found in our two patients is an axial loading of the ipsilateral femur through direct knee impact. This occurred as a result of low energy unprotected forward falls. Significant hip injuries, including fractures and dislocations, have been well documented in high energy trauma from direct anterior knee impact axial loading of the femur [12].

The so-called dashboard knee injury has been studied, usually under the circumstances of motor vehicle accidents and, generally, in a younger population. The fracture tolerance of the hip relative to that of the knee and thigh for knee impact conditions has been particularly investigated. Results show that the hip is the weakest part of the knee-thigh-hip complex when the knee strikes the knee bolster during a frontal vehicle impact, and that hip flexion and adduction from the neutral driving posture significantly reduce hip fracture tolerance [13]. Hip injuries result from the dissipation of a large amount of energy about the hip joint. Clinically, these forces are first transmitted through the knee en route to the hip. There is evidence of substantial forces being directly applied to the knee [12] in these high energy situations.

A recent study by Yamamoto *et al.*[14] clarifies some of the various mechanisms of hip fracture in the elderly and investigates methods of fracture prevention. They developed a fall simulation system and have studied various unprotected falls (slip, trip and faint) that result in direct anterior knee contact. Their results show that a knee grounding fall caused a higher risk of hip fracture than the risk by a lateral impact on the greater trochanter. These findings support the mechanical relationship between the physical finding of knee impact and hip fracture found in the present review and indicate an axial loading mechanism.

## Conclusions

The physical finding of a small localized site of impact and/or contusion in an anterior knee location in both patients with radiologic evidence of an ipsilateral hip fracture would strongly suggest that a knee impact injury can transmit enough energy to the proximal femur by axial loading to result in the hip fracture. This physical finding (Knee Impact Sign*),* described herein, is a reliable indicator of this hip fracture mechanism*.* This sign should be looked for in the evaluation of patients, particularly the elderly, in whom hip trauma or fracture is suspected.

The conclusions reached in this case series in no way conflict with the current hip fracture literature, but add to our understanding of these fractures. We remain in agreement that most hip fractures are the result of a sideways fall with impact occurring at or near the hip. There is an opportunity for further study and future biomechanical investigations to define better the nature and true frequency of the association of this physical sign and hip fractures.

## Consent

Written informed consent was obtained from the patients for publication of this case series and any accompanying images. A copy of the written consent is available for review by the Editor-in-Chief of this journal.

## Competing interests

The authors declare that they have no competing interests.

## Authors’ contributions

DWK treated both patients, obtained the patients’ written informed consent to publish the report, conducted the follow-up examinations, analyzed and interpreted the patient data and wrote and edited the manuscript. BDK reviewed the literature, collected patient data and assisted in final editing. Both authors read and approved the final manuscript.
